# Noticing relevant problem features: activating prior knowledge affects problem solving by guiding encoding

**DOI:** 10.3389/fpsyg.2013.00884

**Published:** 2013-11-26

**Authors:** Noelle M. Crooks, Martha W. Alibali

**Affiliations:** Department of Psychology, University of Wisconsin - MadisonMadison, WI, USA

**Keywords:** mathematics education, problem solving, encoding, equivalence problems, equal sign

## Abstract

This study investigated whether activating elements of prior knowledge can influence how problem solvers encode and solve simple mathematical equivalence problems (e.g., 3 + 4 + 5 = 3 + __). Past work has shown that such problems are difficult for elementary school students (McNeil and Alibali, 2000). One possible reason is that children's experiences in math classes may encourage them to think about equations in ways that are ultimately detrimental. Specifically, children learn a set of patterns that are potentially problematic (McNeil and Alibali, 2005a): the perceptual pattern that all equations follow an “operations = answer” format, the conceptual pattern that the equal sign means “calculate the total”, and the procedural pattern that the correct way to solve an equation is to perform all of the given operations on all of the given numbers. Upon viewing an equivalence problem, knowledge of these patterns may be reactivated, leading to incorrect problem solving. We hypothesized that these patterns may negatively affect problem solving by influencing what people encode about a problem. To test this hypothesis in children would require strengthening their misconceptions, and this could be detrimental to their mathematical development. Therefore, we tested this hypothesis in undergraduate participants. Participants completed either control tasks or tasks that activated their knowledge of the three patterns, and were then asked to reconstruct and solve a set of equivalence problems. Participants in the knowledge activation condition encoded the problems less well than control participants. They also made more errors in solving the problems, and their errors resembled the errors children make when solving equivalence problems. Moreover, encoding performance mediated the effect of knowledge activation on equivalence problem solving. Thus, one way in which experience may affect equivalence problem solving is by influencing what students encode about the equations.

## Introduction

A crucial step in solving problems is noticing features that are relevant for their solution. For example, in solving an arithmetic problem, it is necessary to note whether the operator symbol is a plus, minus, times, or division sign. Misencoding the operator would lead one to enact an incorrect strategy and obtain an incorrect solution. Thus, proper encoding of relevant problem features can mean the difference between solving a problem correctly and getting it wrong. Past research in child development and in adult cognition has highlighted the importance of accurate encoding in a number of domains, including probability (Dean and Mollaison, 1986), spatial mapping (Chen, 2007), proportional reasoning (Fujimura, 2001), and insight problem solving (Kaplan and Simon, 1990).

What factors guide solvers' problem encoding? One important factor is prior knowledge. Learners' prior knowledge may provide them with information about what sorts of problem features are important. In mathematical equations, for example, numbers and operation symbols are important features, whereas other features (e.g., the font in which a problem is printed) can be safely ignored. Evidence from a range of problem domains shows that learners with greater prior knowledge encode key problem features more accurately than learners with less prior knowledge (e.g., Chi et al., 1981; Werner and Thies, 2000; Booth and Davenport, 2013).

A second factor that influences learners' encoding of novel problems is the amount of perceptual overlap between new problems and previously encountered problems (e.g., McNeil and Alibali, 2004). Chase and Simon's (1973) classic study, for example, showed that chess experts were poor at recalling random arrangements of chess pieces, but were quite accurate when the chessboard was arranged in a plausible game configuration. The plausible game configurations presumably had much greater perceptual overlap with familiar configurations. Participants' ability to recall patterns that matched familiar ones was greater than their general recall for information that was perceptually unlike previously seen chess boards.

A third factor that affects problem encoding is the strategy that the solver plans to enact (e.g., Alibali et al., 2009). Features relevant to the planned strategy are encoded, while other features may be overlooked. For example, many young children solve balance scale problems by comparing the relative weights on the two sides of the fulcrum. Children who use this strategy often fail to encode the distance of the weights from the fulcrum (Siegler, 1976).

We suggest that, when presented with a novel problem, solvers activate elements of their existing knowledge, and this activated knowledge guides their problem encoding. For example, when presented with a problem, solvers may activate knowledge of possible problem-solving strategies, because of their past experience with problems of that type. They may also activate knowledge of related problems, due to perceptual overlap of the given problem with related problems. We propose that the specific knowledge that solvers activate should influence their encoding of novel problems, and we test this idea in the present experiment. If the activated knowledge is relevant to the given problem, solvers should accurately encode and solve the given problem. If, on the other hand, the activated knowledge does not align with the given problem, it may be detrimental for encoding and solving.

## Prior knowledge in mathematics

One domain in which the relationship between prior knowledge and current behavior may be particularly important is mathematical problem solving. Mathematics is a cumulative subject, in which new information builds on concepts and procedures that have been encountered previously. Although drawing on prior knowledge is often beneficial, there are some situations in which activating prior knowledge may actually hinder new learning and problem solving.

### Mathematical equivalence

One area of mathematics in which previous experience has been posited to have a substantial negative effect is in equation solving. Elementary and middle school students often fail to correctly solve equations that have operations on both sides of the equal sign (e.g., 3 + 4 + 5 = 3 + __), called *mathematical equivalence problems* (e.g., Perry et al., 1988). Despite having the prerequisite knowledge of addition and subtraction necessary to solve such problems, children continue to have difficulty, even into their later elementary and middle school years (e.g., McNeil, 2007). Poor performance on equivalence problems is problematic for learners, because understanding equality is foundational for later mathematics, particularly algebra (e.g., Falkner et al., 1999; Knuth et al., 2005). Algebra, in turn, has been identified as a “gatekeeper” for later educational and employment success (e.g., Ladson-Billings, 1998; National Research Council, 1998; Moses and Cobb, 2001).

Why do children perform poorly on equivalence problems? One possibility is that the instructional materials and techniques used in elementary education in the United States may lead children to misconstrue the equal sign as an operator (meaning “find the total”), rather than as expressing an equivalence relation. Both elementary and middle school textbooks in the United States typically present the equal sign in a very narrow set of contexts, and many textbooks do not provide explicit instruction about the symbol and its use (e.g., McNeil et al., 2006; Li et al., 2008; Rittle-Johnson et al., 2011).

### Prior arithmetic experience and equivalence understanding

Children's experience during elementary school appears to teach children three patterns—perceptual, conceptual, and procedural—that may hinder their abilities to solve more complex equations (e.g., McNeil et al., 2010). Children are thought to abstract these patterns from their experiences with arithmetic operations. McNeil and Alibali (2005a) argued that these three patterns, termed “operational patterns,” can combine to create an entrenched misunderstanding of mathematical equivalence. When encountering a novel mathematical equation, these three patterns may become activated in the child's mind and guide problem solving. If the novel problem is a traditional arithmetic problem, activation of this knowledge might facilitate problem solving. If, however, the novel problem does not follow the traditional format (e.g., an equivalence problem), activation of this knowledge might be harmful. Indeed, McNeil and Alibali (2005a) showed that not only is knowledge of the three operational patterns associated with poorer performance on equivalence problems, but also that children with deeply entrenched misconceptions are less likely to learn from later direct instruction about solving equivalence problems.

First, children learn the *perceptual pattern*^1^ that equations always follow an “operations = answer” format (e.g., Carpenter et al., 2003). This misconception is often perpetuated in textbooks, with some textbooks showing the equal sign in this format up to 70% of the time (McNeil et al., 2006).

Second, children learn the *conceptual pattern* that the equal sign means to “calculate the total” or to “put the answer.” In fact, when asked to define the equal sign, elementary and middle school students often give operational definitions such as these instead of relational definitions (e.g., “the same as”) (Knuth et al., 2005; McNeil and Alibali, 2005b). Although an operational understanding of the equal sign is sufficient for success on traditional arithmetic problems (e.g., 2 + 2 = __), it does not generalize to problems where a relational understanding is necessary (e.g., equivalence problems, algebraic equations). Middle-school students who give operational definitions when asked to define the equal sign are significantly worse at solving algebra problems than those who give relational definitions (Knuth et al., 2006).

Third, children learn the *procedural pattern* that the correct strategy for solving a math problem is to perform all of the given operations on all of the given numbers. This misconception is reflected in the incorrect answers children typically give when solving equivalence problems, with many students simply adding up all the numbers (i.e., an *add-all* strategy) to get their solution, as if solving a typical arithmetic problem (e.g., Falkner et al., 1999).

Although these patterns are pervasive in American educational settings and curricular materials, they are not universal. Children in countries where the equal sign is presented in more varied contexts, both in textbooks and in classrooms (e.g., China), perform significantly better than American students on equivalence problems, with sixth graders solving up to 98% of problems correctly (Li et al., 2008). It appears that American children's experience with the equal sign during their elementary school education may lead to misconceptions that hinder their ability to solve equivalence problems.

### Effect of activating prior knowledge on equivalence problem solving

In addition to correlational evidence for the relationship between certain types of experience in the mathematics classroom and trouble solving equivalence problems, research with undergraduate participants supports a causal link between activation of the operational patterns and such difficulties (McNeil and Alibali, 2005a). Of course, adults generally reason in more advanced ways than children. However, there is also evidence suggesting that new knowledge and strategies do not necessarily replace old knowledge (e.g., Siegler, 2006; Shtulman and Valcarcel, 2012). Different situations can cause different pieces of knowledge to be activated, leading learners to sometimes rely on advanced ways of thinking and at other times to revert to more naïve approaches (e.g., Munakata et al., 1997; Siegler and Stern, 1998). In mathematical equivalence, for example, undergraduate students who received their early education in the US appear to hold both a sophisticated, relational understanding of the equal sign and a naïve, operational understanding. Seeing the equal sign in different contexts may activate one of these conceptions more strongly than the other, and contexts that activate the operational view sometimes lead undergraduates to define the symbol in the same way that young elementary school students do (McNeil and Alibali, 2005b).

Reactivating knowledge of the operational patterns can also affect how undergraduate participants *solve* equivalence problems. For example, after having all three operational patterns activated with a brief set of tasks, only 42% of undergraduates succeeded in correctly solving at least 7 of a set of 8 equivalence problems, compared with 92% in a control group not exposed to the patterns (McNeil and Alibali, 2005a).

Even simple practice solving traditional arithmetic problems (i.e., those with the equal sign and answer blank at the end of the problem) has been shown to negatively affect undergraduates' ability to solve equivalence problems. McNeil et al. (2010) asked participants either to solve a set of traditional addition problems (e.g., 8 + 4) or to complete a control task. At posttest, participants who had completed the addition problems were significantly worse than control participants at solving both equivalence problems and simple algebra problems. Importantly, the undergraduates in these studies were not only worse at solving the equivalence problems, but they also often used the add-all strategy typically used by young children. These findings are presumably due to the arithmetic task reactivating participants' knowledge of the operational patterns. McNeil and colleagues interpreted their findings as evidence for continuity in the way that children and adults think about equations.

### Encoding of equivalence problems

The studies reviewed above suggest a possible causal link between the activation of the operational patterns and difficulty solving equivalence problems. One question that remains open, however, is exactly *how* these experiences affect students' thinking.

One possibility is that these operational patterns hinder children's performance by affecting what they encode about mathematics problems. If children fail to notice relevant features of a problem's structure (e.g., the location of the equal sign), they are unlikely to correctly solve the problem. Equivalence problems are perceptually very similar to traditional arithmetic problems, with which elementary school children are often highly skilled (e.g., 3 + 4 + 5 = 3 + __ vs. 3 + 4 + 5 + 3 = __). This perceptual similarity, combined with the operational patterns children have learned, may activate children's arithmetic knowledge, leading them to focus on features that are relevant for solving a traditional arithmetic problem (e.g., the numbers) as opposed to those that are relevant for solving a more complex equation (e.g., the location of the equal sign).

Indeed, when children are asked to reconstruct equations after a brief viewing, they make significantly more errors on blank-final equivalence problems (e.g., 3 + 4 + 5 = 3 + __), which are perceptually highly similar to traditional arithmetic problems, than on non-blank-final equivalence problems (e.g., 3 + 4 + 5 = __ + 5), which are visually less similar (McNeil and Alibali, 2004). In fact, of students who made errors on the blank-final equivalence problems, 58% of them reconstructed at least one problem as if it were a traditional arithmetic problem. Activating solvers' knowledge of basic arithmetic may make the perceptual similarities between traditional problems and equivalence problems highly salient, while also drawing attention away from the equal sign.

Activating solvers' knowledge of basic arithmetic may also activate a problem-solving strategy such as “perform all given operations on all given numbers”, which is commonly used in arithmetic. When applied to an addition equivalence problem, this strategy is implemented as the *add-all* strategy (e.g., leading to the incorrect solution of 15 for the problem 3 + 4 + 5 = 3 + __). Planning to enact this strategy may lead to solvers to encode problems as traditional arithmetic problems, and may lead to errors that are consistent with this planned strategy (e.g., reconstructing 3 + 4 + 5 = 3 + __ as 3 + 4 + 5 + 3 = __).

In sum, there is growing evidence that activation of the operational patterns typical of early arithmetic experience can negatively affect equivalence problem solving. There is also correlational evidence showing that children who have difficulty solving equivalence problems tend to have difficulty encoding them. To date, however, there is no evidence for a causal link between activation of the operational patterns and poor encoding of equations.

## Current study

The purpose of the current study was to test whether activation of the operational patterns hinders problem solving by changing what solvers encode about the problems. Although accurate encoding is a prerequisite for problem solving, there are other ways in which students could err when solving an equation. For example, experience with one problem solving procedure (e.g., *add-all*) could create a mental set, such that students continue to execute a well-practiced strategy, even when it is no longer appropriate (e.g., Luchins, 1942). This could occur even when students encode problems correctly. Thus, it is important to test the possible causal pathway between activation of operational arithmetic knowledge, encoding, and problem solving. As a secondary goal, we sought to replicate past findings demonstrating a link between the operational patterns and difficulty solving problems that do not adhere to a traditional arithmetic format.

In the present study, we utilized the knowledge activation paradigm developed by McNeil and Alibali (2005a) to test whether activating the operational patterns affects solvers' abilities to accurately encode, as well as correctly solve, equivalence problems. To test this hypothesis in children would require strengthening their naïve conception of the equal sign, and we believe this could have negative consequences for their mathematical development. Therefore, for ethical reasons, we chose to test this hypothesis in undergraduate participants. This approach has been used in previous work, both on mathematical equivalence (e.g., McNeil et al., 2010) and in other mathematical domains (e.g., Brunstein et al., 2009). Experimental evidence from undergraduate participants can supplement the correlational data from children by testing a causal link between activation of the operational patterns and difficulty encoding and solving equivalence problems.

Participants in this study were randomly assigned to either a knowledge activation condition or a control condition, and were then asked both (a) to reconstruct equivalence problems after viewing them briefly (to assess encoding) and (b) to solve equivalence problems. We hypothesized that activation of the operational patterns would affect not only participants' solutions to the equivalence problems, as shown in past work, but also their encoding of the problems. Moreover, we hypothesized that inaccurate encoding would mediate the effect of knowledge activation on problem solving. Finally, we expected that activation of the operational patterns would lead participants to encode and solve the equations as if they were traditional arithmetic problems.

## Methods

### Participants

Participants were 181 undergraduate students recruited from the Introduction to Psychology class participant pool at a large Midwestern university. Because we were interested in reactivating the operational patterns that are typical in American educational settings, we limited the sample to participants who were educated in the US (*n* = 140). Additionally, as overall mathematics ability was used as a covariate in our analyses, we excluded participants who did not report quantitative standardized test scores (*n* = 24). One additional participant was excluded from analysis for not having taken a math class within the last 20 years. Thus, the final sample consisted of 115 undergraduates (54% female) who ranged in age from 18–24 years (*M* = 18; 10). The sample was 84% Caucasian, 9% Asian, 3% African American, 2% Hispanic or Latino, and 2% of other racial or ethnic background. On average, participants had SAT or ACT math scores in the 89th percentile (range: 25th–99th percentile).

For the problem-solving task, a subset of participants failed to follow instructions and was therefore excluded from analyses of that task. Specifically, these participants continued to perform the previous task (reconstruction) even after they received instructions to switch to the next task (problem solving). Thus, for problem-reconstruction analyses, the sample consisted of the entire 115 participants and, for the problem-solving analyses, the sample consisted of 100 participants. The number of excluded participants did not differ by condition.

The current work was approved by and conducted in accordance with the human subjects guidelines of the University of Wisconsin-Madison Social and Behavioral Science Institutional Review Board (IRB). Informed consent was obtained from all participants and a debriefing was provided at the conclusion of the experiment. Participants received one point of extra credit in their Introduction to Psychology class in exchange for their participation.

### Knowledge activation and control tasks

Participants completed tasks, adapted from McNeil and Alibali (2005a), that either activated the three operational patterns (knowledge activation tasks) or that involved similar activities without activating the patterns (control tasks) (see Appendix). All knowledge activation and control tasks were completed on a computer.

#### Perceptual pattern activation

Participants saw a target stimulus at the top of the screen followed by five sample stimuli. They were asked to indicate whether or not each of the sample stimuli matched the target, and to respond by pressing a button on the keyboard. In the knowledge activation version of this task, stimuli were equations presented in the “operations = answer” format (e.g., 365 + 694 = __), so as to activate the perceptual pattern. In the control version of the task, stimuli were letter strings (e.g., XxCxcX).

#### Conceptual pattern activation

Participants were shown a target word at the top of the screen followed by five sample words. They were asked to indicate whether each of the sample words matched the target, and to respond via button press. In the knowledge activation version of the task, the words (i.e., total, add, sum, and plus) were selected to activate the concept of “the total.” In the control version of the task, the words were neutral, non-mathematical words (e.g., apple).

#### Procedural pattern activation

Participants saw a target stimulus at the top of the screen followed by five sample stimulus pairs. Participants were asked to indicate whether each of the sample pairs would combine to make the target. In the knowledge activation version of the task, the targets were numbers (e.g., 17) and the sample pairs were sets of numbers (e.g., 8 and 9), so as to activate the strategy of performing all of the given operations on all of the given numbers. In the control version, the targets were colors (e.g., pink) and the sample pairs were sets of colors (e.g., red and white).

### Assessing encoding

To assess their abilities to accurately encode equivalence problems, participants completed a problem reconstruction task. Participants viewed four equivalence problems, one at a time, on a computer screen. Problems were presented for 1.5 s each and after each problem, participants were asked to write the equation exactly as they saw it on a paper answer sheet. Reconstruction tasks have been used extensively in the literature to assess encoding, both in the domain of mathematical equivalence (e.g., Rittle-Johnson and Alibali, 1999) and in other domains (e.g., Chase and Simon, 1973; Siegler, 1976; Booth and Davenport, 2013).

### Assessing problem-solving performance

To assess their abilities to correctly solve equivalence problems, participants completed a problem-solving task. Participants viewed four equivalence problems, one at a time, on a computer screen. Problems were shown for 1.5 s each and participants were then asked to write the number that should go in the blank on a paper answer sheet.

### Procedure

Participants were randomly assigned to either the knowledge activation condition or the control condition. The samples in the two conditions did not differ in terms of gender, age, or percentile SAT/ACT scores.

In the knowledge activation condition, participants performed one block each of the three activation tasks. In the control condition, participants performed one block each of the three control tasks. Tasks were presented in a fixed order (perceptual, conceptual, and procedural) that did not vary by condition, following the procedure used by McNeil and Alibali (2005a). Each block consisted of eight targets, each followed by five samples, presented one at a time, requiring participants to make 40 judgments for each task.

Following the knowledge activation or control tasks, participants completed an assessment comprised of the problem reconstruction task and the problem-solving task. Because we expected that effects of knowledge activation might dissipate quickly, half of the participants completed the problem reconstruction task first and half completed the problem-solving task first. The order of problems within each assessment task was fixed (see Table 1).

**Table 1:** **Problems for problem reconstruction and problem-solving tasks**.

**Task**	**Problem**
Problem reconstruction	3 + 4 + 9 = 3 + __
Problem reconstruction	5 + 8 + 7 = 5 + __
Problem reconstruction	9 + 6 + 3 = 9 + __
Problem reconstruction	6 + 7 + 3 = 6 + __
Problem solving	4 + 3 + 6 = 4 + __
Problem solving	3 + 9 + 5 = 3 + __
Problem solving	7 + 5 + 4 = 7 + __
Problem solving	8 + 4 + 6 = 8 + __

### Coding

#### Problem solving

Problem solutions were coded as either correct or incorrect. The problem-solving test included 4 problems, so participants could receive total scores from 0–4. Additionally, incorrect solutions were further coded in terms of the specific strategy used, as inferred from the solution, based on a scheme developed by Perry et al. (1988), as seen in Table 2.

**Table 2:** **Incorrect strategies for solving the equivalence problem 3 + 4 + 5 = __ + 5**.

**Strategy**	**Solution**	**% of Errors**
Add all	17	37
Add to equal	12	5
Idiosyncratic	2	43
No response	NA	15

#### Problem reconstructions

Participants' reconstructions were coded using the system developed by McNeil and Alibali (2004). Each reconstruction was coded as either correct or incorrect. Incorrect reconstructions were further coded to indicate the type of error made by the participant. *Number* errors involved inaccurately reconstructing the numbers in the problem or their order. *Conceptual* errors involved inaccurately reconstructing the structure of the problem, as seen in Table 3. Note that reconstructions could be coded as errors even if they were valid equations, as the task was to reconstruct the original equation. The reconstruction test included 4 problems, so participants could receive scores from 0–4, based on the number of reconstructions that were free from conceptual errors. In our analysis, we focused on conceptual errors because equivalence problems differ from traditional arithmetic problems in their structure. However, the pattern of results does not differ if total number of errors is used as the dependent variable.

**Table 3:** **Examples of conceptual errors in reconstructing the equivalence problem 3 + 4 + 5 = __ + 5**.

**Reconstruction**	**Error code**	**Explanation**	**% of Errors**
3 + 4 + 5 + 5 = __ 3 + 4 + 5 + 5	Add all	Structure changed to standard addition problem, equal sign may be present or not	50
3 + 4 + 5 = __ 3 + 4 + 5	Add to equal	Structure changed such that the right side is omitted, equal sign may be present or not	9
3 + 4 = __	Add two	Structure is changed such that only the first two addends appear	7
3 + 4 + 5 = 5	No right plus, No blank	Reconstruction lacks a right plus sign and a blank	15
Varied	Idiosyncratic	Reconstruction does not fall into another category	19

## Results

### Problem encoding

**Figure 1:**
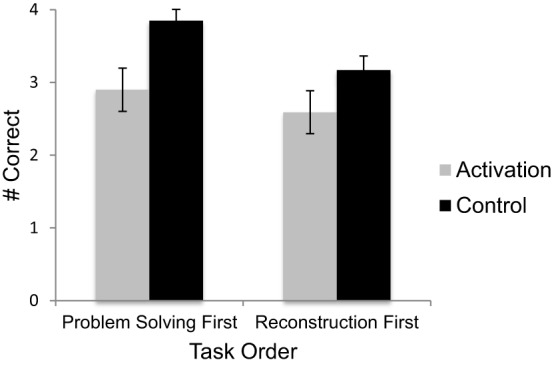
**Performance on the problem reconstruction task by task order and condition**. Error bars represent ±1 standard error.

We predicted that participants whose knowledge of the operational patterns was activated would encode equivalence problems less accurately than control participants. We tested this prediction using a Two-Way (condition x order) between-subjects ANOVA, with percentile SAT/ACT math score as a covariate. As predicted, participants in the knowledge activation condition correctly reconstructed fewer problems (*M* = 2.71) than participants in the control condition (*M* = 3.53), *F*_(1, 110)_ = 11.96, *p* = 0.001, η^2^_*p*_ = 0.098 (see Figure 1). Moreover, participants in the knowledge activation condition were significantly more likely to reconstruct problems as if they were traditional arithmetic problems (*M* = 0.65) than were control participants (*M* = 0.13), *F*_(1.110)_ = 11.19, *p* = 0.001, η^2^_*p*_ = 0.09. No other types of reconstruction errors were affected by condition. There was also a significant effect of task order, *F*_(1, 110)_ = 4.00, *p* = 0.048, η^2^_*p*_ = 0.04; participants who completed the encoding task first correctly reconstructed fewer equations (*M* = 2.89) than participants who solved problems first (*M* = 3.36) (see Figure 1). There was no significant condition by order interaction. Lastly, percentile SAT/ACT math scores were associated with encoding performance, *F*_(1, 110)_ = 11.43, *p* = 0.001, η^2^_*p*_ = 0.094. Overall, participants with higher scores tended to reconstruct more problems accurately.

### Problem solving

**Figure 2:**
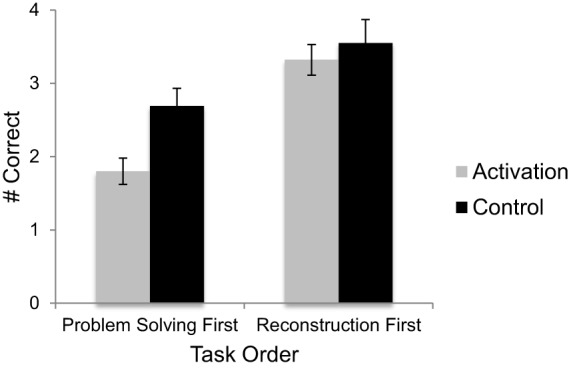
**Performance on the problem solving task by task order and condition**. Error bars represent ±1 standard error.

We also predicted that participants in the knowledge activation condition would be worse at solving equivalence problems than control participants. We tested this prediction using a Two-Way (condition x order) between-subjects ANOVA, with percentile SAT/ACT math score as a covariate. As predicted, participants in the knowledge activation condition solved significantly fewer problems correctly (*M* = 2.53) than did participants in the control condition (*M* = 3.15), *F*_(1, 95)_ = 6.25, *p* = 0.014, η^2^_*p*_ = 0.062 (see Figure 2). Not only were participants in the knowledge activation condition worse at solving the problems, they also made significantly more add-all errors (*M* = 0.61) than did control participants (*M* = 0.09), *F*_(1, 95)_ = 7.54, *p* = 0.007, η^2^_*p*_ = 0.07. No other incorrect strategy types were affected by condition. There was also a significant effect of task order, *F*_(1, 95)_ = 23.91, *p* < 0.001, η^2^_*p*_ = 0.20; participants who completed the problem solving task first solved significantly fewer problems correctly (*M* = 2.23) than participants who completed the encoding task first (*M* = 3.45) (see Figure 2). There was no significant condition by order interaction. Finally, percentile SAT/ACT math scores were associated with problem solving performance, *F*_(1, 95)_ = 10.16, *p* = 0.002, η^2^_*p*_ = 0.097. Overall, participants with higher SAT/ACT math scores tended to solve more problems correctly.

### Mediation analysis

**Figure 3:**
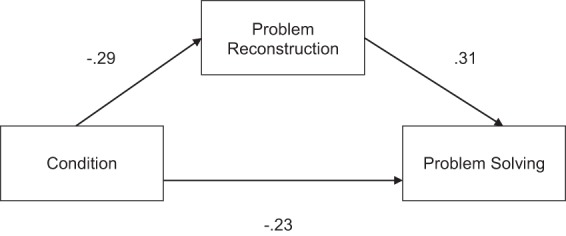
**Mediation analysis of the effect of condition on problem solving**. Standardized regression coefficients (ß) are reported.

We next sought to test whether the relationship between knowledge activation and problem solving performance could be explained by decreased accuracy in problem encoding. To do so, we conducted a mediation analysis (Baron and Kenny, 1986). As predicted, encoding mediated the relationship between activation of the operational patterns and problem-solving performance, as shown by a significant Sobel test, *z* = 2.43, *p* = 0.008 (see Figure 3). Adding problem reconstruction to the model led to a 43% reduction in the proportion of variance accounted for by condition (ß = −0.23 vs. ß = −0.13).

## Discussion

In this experiment, we tested a hypothesized causal link between activation of the operational patterns and inaccurate encoding of mathematical equivalence problems. No previous studies have provided causal evidence for this connection. To obtain such evidence, we examined the effects of activating knowledge of the three operational patterns on undergraduate students' encoding of mathematical equivalence problems. We also examined effects on problem solving.

### Did activating the operational patterns affect problem encoding and solving?

As predicted, activating knowledge of the operational patterns led participants to incorrectly reconstruct the equations, suggesting that being induced to think about the patterns affected what they noticed and encoded. Not only were participants in the knowledge activation condition more likely to make reconstruction errors, they were also more likely to encode the equivalence problems as if they were traditional arithmetic problems. These findings resemble the patterns found in children, in which knowledge of the operational patterns correlates with difficulties reconstructing equivalence problems (McNeil and Alibali, 2005a). It appears that one way in which early arithmetic experience may hinder performance on equivalence problems is by promoting inaccurate encoding, specifically, misencoding of such problems as traditional arithmetic problems.

The current findings also replicated previous work demonstrating that experience with the operational patterns hinders equivalence problem solving (McNeil and Alibali, 2005a; McNeil et al., 2010). As predicted, exposure to the operational patterns had a significant negative effect on problem solving performance. Not only were participants in the knowledge activation condition worse at problem solving, but they also performed similarly to children; when faced with an equivalence problem, they were more likely to solve it like a traditional arithmetic problem by simply adding all the numbers.

Our mediation analysis supports the claim that poor encoding is one mechanism by which exposure to the operational patterns negatively affects problem solving. Specifically, we found that the relation between condition and problem-solving performance was partially mediated by performance on the problem reconstruction task. Thus, knowledge activation affected problem solving, at least in part, by affecting encoding. However, it is worth emphasizing that the mediation we observed was partial, rather than complete; thus, there are likely other mechanisms at play as well.

### Other factors that affected encoding and problem solving

In general, participants who performed better on standardized tests of mathematics were significantly better both at encoding and solving equivalence problems, regardless of condition. This finding was not surprising, as previous work with middle-school students has demonstrated that students who perform better on standardized tests of mathematics are more likely to have a relational understanding of the equal sign (Knuth et al., 2006). At present, we cannot discern whether the effects we observed were due to differences in underlying mathematical ability or differences in mathematical experience. In our sample, participants with higher standardized test scores also tended to have taken more advanced mathematics courses, which may have given them additional experience with complex and non-traditional problem formats. Thus, these findings are also compatible with previous work demonstrating an effect of educational experiences on understanding of mathematical equivalence (McNeil and Alibali, 2005b).

We also found that the effect of knowledge activation was fleeting—it was always strongest in the first task following the activation tasks. Thus, the order of the tasks affected participants' encoding and problem solving. Because the activation phase of the current study lasted only a few minutes, it is not surprising that the performance decrements began to fade quickly. It is also possible that the effects of knowledge activation might last different lengths of time for participants with different levels of mathematical knowledge (McNeil et al., 2010). Participants with lower levels of math knowledge or experience may have stronger operational conceptions and be more susceptible to knowledge activation, or their operational conceptions, once activated, may take longer to return to baseline levels of activation. Although some participants in our sample had taken more math classes than others, they were all college educated and thus there was not sufficient variability in mathematics knowledge in our sample to test these predictions. Future work should explore the effects of knowledge activation in learners with varying levels of education to test the power and duration of the effect. Despite the short-lived nature of our intervention, our data do indicate that even brief exposure to the operational arithmetic patterns can affect performance on equivalence problems, even in participants for whom these problems should be quite simple.

It is worth noting that the present study utilized control tasks that were non-mathematical in nature (i.e., strings of letters for the perceptual control, concrete words for the conceptual control, and combining colors for the procedural control). In light of other work (e.g., McNeil et al., 2010), we believe that the findings would have been similar if had we used control tasks that were mathematical but not arithmetic based. However, it is also possible that mathematical control tasks could activate other elements of solvers' prior knowledge that could affect their performance in systematic ways. Future research is needed to more fully understand the connections among elements of mathematical knowledge, and how activating one element leads to activation of related elements.

### How does each operational pattern independently affect encoding and problem solving?

The current study suggests that activation of the operational arithmetic patterns may hinder problem solving by negatively affecting problem encoding. However, in the present study we could not assess the unique effects of each of the operational patterns on encoding. Prior knowledge could influence encoding in a number of ways, and it may be that the different operational patterns affect what participants notice in different ways. For example, the perceptual pattern of the “operations = answer” format may affect encoding because of the high level of perceptual similarity between traditional arithmetic problems and equivalence problems (e.g., McNeil and Alibali, 2004). The procedural pattern, on the other hand, may affect encoding by leading problem solvers to focus only on features relevant for the strategy they plan to enact (e.g., adding up all the numbers to find the “total”) (e.g., Siegler, 1976). It may also be that only some of the operational patterns affect encoding, whereas the others affect behavior through different mechanisms or have no effect. The procedural pattern, for example, may simply create a mental set such that participants continue to utilize a highly practiced strategy, even after it ceases to be effective (e.g., Luchins, 1942). We are addressing these issues in ongoing work that investigates the unique effects of each operational pattern. Separating out the unique ways in which each of the operational patterns influences performance will provide a more nuanced understanding of the effects of different types of arithmetic experience on equivalence problem encoding and solving. With a better understanding of the causal mechanisms involved, we will be in a better position to make recommendations about how to restructure children's early mathematics education in order to avoid common problems.

### Implications

In considering the implications of this research, it is important to bear in mind that we studied adult participants in order to shed light on the mechanisms underlying a phenomenon typically found in children. We chose to study adults because we believe it would be unethical to purposely expose child participants to information that we expect to have negative effects on their learning. Work with adults can provide corroborating evidence for our hypotheses without potential harm to young students.

The current findings help build the case that knowledge of the operational patterns *can* negatively affect encoding and solving of equivalence problems. However, this does not necessarily establish that this *is* the mechanism at play in children. Our experiment utilized a brief manipulation in a laboratory context with adults and thus is far removed from the elementary mathematics experience of children. We do not intend to imply that activating knowledge of the operational patterns via experimental tasks (as we have done with adults) yields the same knowledge state as building knowledge of operational patterns through experience with arithmetic instruction and practice (as occurs in children). However, previous work has demonstrated continuity in equation understanding between children and adults (McNeil et al., 2010), suggesting that knowledge of the operational patterns that American children learn in their early education remains through adulthood. While undergraduates typically utilize their newer, relational understanding of equivalence, certain situations may cause them to revert back to more naïve approaches—and our knowledge activation paradigm was one such situation. The fact that adults in our study made the same types of errors that children typically make lends plausibility to this hypothesized causal pathway.

We suggest that curricular activities and homework assignments that emphasize the operational patterns may lead children to activate knowledge of those patterns. Thus, it seems reasonable to argue that, in children's day-to-day mathematics experience, they often activate the operational patterns, and those patterns affect their encoding and solving of equations.

The present findings also highlight the importance of problem encoding in correctly solving equations. Thus, our data underscore previous work suggesting that guiding students to notice the appropriate features of mathematical inscriptions might foster their understanding (e.g., Lobato et al., 2003). Specifically, we suggest that one potentially effective educational intervention may involve guiding children to focus on the problem features that are relevant in a given situation. For example, in equivalence problems, where the equal sign is often misencoded, children who have their attention drawn to the equal sign show improvements in both problem encoding and problem solving (e.g., Alibali et al., under review).

Our findings also suggest that it may be wise to teach children arithmetic in ways that do not entrench the operational patterns. Recent work by McNeil et al. (2011) has shown that teaching arithmetic in a way that elucidates the meaning of the equal sign may help prevent later difficulties in understanding equations.

## Conclusion

In sum, the current work adds to a growing body of literature emphasizing the effects of prior knowledge on later mathematical performance. In this study, we found that activating adults' knowledge of the three operational patterns common in arithmetic led to difficulties in their encoding and solving of equations. Moreover, poor encoding partially mediated the relationship between activation of the operational patterns and problem-solving performance, suggesting a mechanism by which knowledge activation might affect success in solving equivalence problems. Specifically, prior knowledge of the operational patterns affects equivalence problem solving by guiding solvers to encode the problems inaccurately. These findings help build the case that experiences with the operational patterns are a potential source of children's persistent difficulties with mathematical equivalence.

## Author contributions

Noelle M. Crooks designed and conducted the experiment, coded and analyzed the data, and wrote the manuscript. Martha W. Alibali assisted in experimental design, analysis, and writing.

### Conflict of interest statement

The authors declare that the research was conducted in the absence of any commercial or financial relationships that could be construed as a potential conflict of interest.

## Footnotes

^1^Note that, while each of the patterns includes perceptual, conceptual, and procedural elements, their names reflect the primary components of each pattern.

## Appendix: activation and control tasks

**Perceptual Tasks**

          **Activation Instructions**. “You will briefly see a target equation at the top of the screen, followed by 5 sample equations in the middle of the screen. If the sample equation is identical to the target, press 1. If the sample equation is not identical to the target, press 2.”

          ***Example Activation Stimuli***

Target: 375 + 695 = ___        Sample: 365 + 694 = ___

          **Control Instructions**. “You will briefly see a target letter string at the top of the screen, followed by 5 sample equations in the middle of the screen. If the sample letter string is identical to the target, press 1. If the sample letter string is not identical to the target, press 2.”

          ***Example Control Stimuli***

Target: XxxxX                   Sample: XcccX

**Conceptual Tasks**

          **Instructions**. “You will briefly see a target word at the top of the screen, followed by 5 sample words in the middle of the screen. If the sample word is identical to the target, press 1. If the sample word is not identical to the target, press 2.”

          ***Example Activation Stimulus***

Target: Total                   Sample: Sum

          ***Example Control Stimulus***

Target: Apple                   Sample: Banana

**Procedural Tasks**

          **Activation Instructions**. “You will briefly see a target number at the top of the screen, followed by 5 sample number pairs in the middle of the screen. If the sample number pair combines to make the target, press 1. If the sample number pair does not combine to make the target, press 2.”

          ***Example Activation Stimulus***

Target: 16                     Sample: 8 and 7

          **Control Instructions**. “You will briefly see a target color at the top of the screen, followed by 5 sample color pairs in the middle of the screen. If the sample color pair combines to make the target, press 1. If the sample color pair does not combine to make the target, press 2.”

          ***Example Control Stimulus***

Target: Orange                   Sample: Yellow and blue
